# High spatial pair cohesion during and after breeding in a socially monogamous territorial passerine

**DOI:** 10.1093/beheco/araf130

**Published:** 2025-11-06

**Authors:** Frigg J D Speelman, Chris W Tyson, Marc Naguib, Simon C Griffith

**Affiliations:** School of Natural Sciences, Macquarie University, 205A Culloden Road, NSW 2113, Australia; Groningen Institute of Evolutionary Life Sciences, University of Groningen, Nijenborgh 7, Groningen 9747AG, The Netherlands; Behavioural Ecology Group, Wageningen University & Research, P.O. Box 338, Wageningen 6700AH, The Netherlands; Behavioural Ecology Group, Wageningen University & Research, P.O. Box 338, Wageningen 6700AH, The Netherlands; School of Natural Sciences, Macquarie University, 205A Culloden Road, NSW 2113, Australia; School of Biological, Earth & Environmental Sciences, University of New South Wales, NSW 2052, Australia

**Keywords:** automated radio tracking, coordination, movement, non-breeding, partnerships, territorial

## Abstract

Long-term social monogamy, a prevalent mating system in avian species, is often associated with increased cooperation and coordination as well as reduced sexual conflict. Although many studies have highlighted the benefits of long-term partnerships for individuals, there remains a lack of insight into how closely partners associate with one another behaviorally. To date, studies investigating pair cohesion in seasonal and long-term partnerships are typically restricted to arrivals at the nest or feeding sites during the breeding season. Using fine-scale automated tracking data on chirruping wedgebills (*Psopodes cristatus*), a territorial socially monogamous species, we characterized how partners coordinate their movement during and after the breeding season. We used 12 pair-bonded individuals with consistently high localization rates that were tracked for a period between 32 and 69 days, with an average of 260,000 localizations per individual. We demonstrate that pairs (1) had extremely similar home ranges with a similarity index of 0.93 versus 0.18 for non-pairs, (2) maintained consistently closer proximity than expected from movement without paying attention to a partner, and (3) followed each other as they moved, with individuals following their moving partner in 42% of cases during and in 47% of cases after breeding. Our findings show that pair cohesion in socially monogamous territorial species can be very high in both a breeding and non-breeding context, illustrating that strong coordination among partners has important functions beyond reproduction and parental care.

## Teaser

Many birds form lasting partnerships, suggesting that spending time with one's partner is very beneficial. Still, we know surprisingly little about their close associations. We studied the movement of chirruping wedgebills and found breeding partners used almost the exact same areas, were consistently close to each other, and follow each other frequently during and after breeding. Overall, being with one's partner is important beyond reproducing together, and partnerships go beyond the requirements for current reproduction.

## Introduction

In socially monogamous species, pair bonds can persist over just one breeding attempt, multiple breeding attempts and even entire lifetimes, which can have fitness consequences such as increased survival (Culina et al. 2015; Jankowiak et al. 2018) and reproductive success (Adkins-Regan and Tomaszycki 2007 ; Sánchez-Macouzet et al. 2014 ; D’Amelio et al. 2024). Although findings on the fitness benefits of persistent partnerships in long-lived species are widespread, there is still a predominant focus on sexual conflict between partners rather than cooperation among individuals reproducing together (Griffith 2019). When attempting to understand the evolution of social partnerships, the coordination within a partnership may be considerable and reduces sexual conflict since the evolutionary interest of both individuals are more aligned (Mariette and Griffith 2015; Patrick et al. 2020). Here, the degree and type of coordination as well as the strength of the association are crucial factors. Association strength is dependent on the decisions made by both individuals forming a dyad (such as a pair bond), and in turn, behavioral decisions depend on the association strength of a dyad (Cantor et al. 2021).

Decision-making processes within a dyad that influence social cohesion are closely linked to space use, as mobile animals must determine when and where to move. Thus, quantifying fine-scale individual movement across space and time in relation to their partner can reflect the strength of the pair association and even the level of cooperation. For example, work on nest-visitation rates of pairs in avian species has demonstrated reproductive benefits of arrival synchrony and provisioning coordination (Mariette and Griffith 2012; Bebbington and Hatchwell 2016; Tyson et al. 2017; Wojczulanis-Jakubas et al. 2023), but this only captures a small proportion of individual space-use. Pair coordination away from the nest has been scarcely investigated due to limitations in monitoring very fine-scale movement of multiple free-ranging and mobile animals across long periods of time in the wild. A recent exception is a study on zebra finches (*Taeniopygia castanosis*), a non-territorial species, where individuals were tracked continuously up to 29 days during a breeding period, showing extremely high overlap in home ranges and consistently high spatial proximity of pairs (Tyson et al. 2024a). Although this shows pairs maintain close spatiotemporal coordination, it is unclear whether spatial pair cohesion is high (1) during the nonbreeding period and (2) in territorial species (beyond nest attendance).

To date, we are aware of no studies on continuous pair movement coordination beyond offspring provisioning and on territorial species specifically. However, movement not directly related to parental care and whether a species is territorial are crucial aspects to consider when assessing social behavior in highly mobile animals. As movement is more restricted and more organized between individuals in territorial species, territoriality has implications for spatial cohesion across individuals, pair-bonded or not, within a population. Previous studies showed the degree of territoriality and territory size is inherently linked to social and reproductive behaviors in many species (see Snijders and Naguib 2017), as it dictates which individuals encounter one another and at what rate. For example, in great tits (*Parus major*), larger home-ranges are associated with higher tolerance against intruders (Naguib et al. 2022), and higher population densities result in stronger territorial responses to simulated intruders (Araya-Ajoy and Dingemanse 2017). Additionally, territoriality may be more effective when a territory owner is more social. For example, male crested titmice (*Baeolophus atricristatus*) attack highly threatening intruders more often when supported by juveniles (Borger et al. 2020). Territorial species also tend to increase their home range sizes when conditions become unsuitable for breeding, such as Bonelli's eagles (*Aquila fasciata*; Bosch et al. 2010) and superb fairy wrens (*Malurus cyaneus*; Camerlenghi et al. 2022). Importantly, to our knowledge, no studies to date have quantified movement coordination and home range overlap of partners during and after breeding.

Here, we quantify the movement patterns and level of spatial cohesion within and among pairs both in and after the breeding season in the chirruping wedgebill (*Psopodes cristatus*), a highly mobile and free-ranging animal that breeds in pairs opportunistically throughout the year in the Australian outback. Although little is known about the breeding ecology of these birds, field observations confirm that chirruping wedgebill pairs tend to stay together between and across breeding seasons (this study), similarly to the closely related Eastern whipbird (*Psophodes olivaceus*; Rogers and Mulder 2004). Chirruping wedgebills are known to produce duets in pairs throughout the year, suggesting pairs may be closely associated and territorial (Austin et al. 2019), and indeed duetting species often form long term pair bonds and are territorial (Tobias et al. 2016). We used an automated radiotracking system including hybrid (solar- and battery-powered) radio tags to enable continuous long-term tracking of territorial pairs and their neighbors. First, we tested whether or not home range size changed across time (daily and weekly) regardless of pair-bondedness, characterizing the territoriality of this species. To determine pair cohesion, we tested whether partners (1) had a larger home-range overlap than dyads that are not pair-bonded, (2) were in close proximity to one another, and (3) tended to follow each other as they move. We predicted that both pair members stay together most of the time, moving as a pair through their home-range. For a species with long-term partnerships and opportunistic timing of breeding we further predicted that social cohesion would persist beyond breeding.

## Methods

### Study species and data collection

Fieldwork was conducted at Gap Hills (30°56′58″S, 141°46′02″E), Fowlers Gap Research Station, New South Wales, Australia, from August to November 2023. Here, we monitored a local population of chirruping wedgebills, a passerine endemic to the Australian outback. We first monitored the population to establish where socially monogamous pairs reside in supposed territories based on locations where individuals forage and produce vocalizations together during the morning (up to 4 hours after sunrise), as well as based on territorial aggressive displays (chasing, attacking, and producing aggressive vocalizations in response to an intruder). Chirruping wedgebills produce sex-specific vocalizations and duet when pair-bonded (Austin et al. 2019), meaning we could identify breeding pairs through consistent duetting of male-female dyads every morning from their roost tree. All target breeding pairs were monitored at least once every two days for breeding behavior (collecting nest material, incubating, provisioning chicks or fledglings) throughout the study period to ascertain the breeding status of individuals and the population.

Once target breeding pairs were identified, we captured these pair-bonded chirruping wedgebills using mist-nets near their roost trees. All birds were banded using an ABBBS metal ring and three color rings, blood-sampled (∼10 µl) via brachial venipuncture for molecular sexing (see below) and tagged with a solar-powered radio tag including a battery and a nylon-coated braided steel antenna (Cellular Tracking Technologies *HybridTag,* New Jersey, USA). Tags were attached using a nylon leg-loop harness, totaling to a maximum of 1.3 g (≤ 3% of body mass). Banded individuals were monitored for a two-week period post-catching to ensure they were not hindered by the radio tag and to verify pair-bonds and territories previously established. When revisiting the field site in February and September 2024, all banded individuals that were resighted (*N* = 16) remained with the same partner within their home ranges, suggesting they form long-term pair bonds.

Blood samples were used for individual sexing by extracting DNA and then using PCR to amplify the CHD locus which is polymorphic between the sex chromosomes and conserved across bird species (Lee et al. 2010). Molecularly verified sexing was consistent with sexing based on the vocalization behavior of individuals.

### Radio-tracking

We tagged 23 pair-bonded adult chirruping wedgebills, that were tracked for between 34 and 69 days (mean = 60.2 days, ± 12.5 s.d.) in the period from 6th of September to 30th of November 2023 during daylight hours when birds were not roosting. We used an automated radiotracking system covering 1.27 km^2^ already installed at the study site (Tyson et al. 2024a), consisting of an array of 94 radio receivers (Cellular Tracking Technologies Node v2, New Jersey, USA) placed 100 to 150 m apart from one another. Tag identity, received signal strength (RSS, a negative value in decibels where values closer to 0 indicates a stronger signal) and time of detection were recorded when radio receivers detected a signal, and sent to the central station aggregating all detection data. We first calibrated the tags, by determining the RSS-distance relationship. We held six tags at 1.5 m high at 18 set distance intervals between 1 to 200 m from four receivers (at 1, 2, 3, 4, 5, 7.5, 10, 12.5, 15, 20, 25, 30, 40, 50, 75, 100, 150, 200 m) and determined the RSS for each distance. RSS values were modeled as a function of Euclidian distance from each receiver: distance (m) ≈ 10^−1.27009 − 0.03302^  ^×^  ^RSS^ (Figure S1; see Tyson et al. 2024a). We also verified how much the elevation of the bird above the ground affected detections by the receivers by mounting a total of 6 tags horizontally on 3 poles, one 10 cm above ground and one 200 cm above ground for each pole. Then, we held these poles at a total of 86 test points (26, 27, and 33 test points per pole) for 2 minutes at a time at uniformly distributed locations within the receiver array. At each test point, we determined the rate of detection, number of receivers picking up the tag, the mean and the maximum RSS for each tag. And tested whether there were significant differences using a Wilcoxon test.

Locations were determined within a 15-second window using two methods: (1) RSS-based multilateration and (2) based on the receiver with the strongest detection of the tag. For the first method using multilateration, we filtered windows in which at least three receivers detected the tag within an interval. We removed windows when the strongest detection had an RSS less than −80 dB (corresponding to a radius of 23.5 m around a radio receiver) to prevent inclusion of inaccurate localizations. Then, we fit a non-linear least-squared model to estimate the location (see Paxton et al. 2022) 100 times sampling around ca. 1 SE around the mean distance for an RSS value (see Tyson et al. 2024a). This yielded an error ellipse corresponding to the square root of two-sigma ellipse of a bivariate normal distribution, representing the level of uncertainty around each localization. Previous field calibrations (Tyson et al. 2024a) found a median difference of 35 m between the estimated and true coordinates of a tag. For the second, simpler, method using the strongest detection of the tag (hereafter “strongest detection method’), we estimated that the location of the tag corresponded to the location of the receiver with the strongest detection, and applying a signal cut-off (RSS ≥ −80 dB) meaning that all detections considered were within a 23.5 m radius of the receiver.

To assess the effectiveness of both methods for continually tracking individuals, assuming that they stayed within the area of the grid, and that the tags were emitting a signal every 15 seconds, we determined the total number of 15-second signals during daylight hours for which the tagged bird could be localized and calculated the percentage of realized localizations during this time period. Then, we estimated the expected percentage of time intervals with detections that birds could be localized using the “strongest detection method”. This method will only detect an individual when it is found within ca. 23.5 m of a receiver assuming a tag height of 1.5 m. Therefore we compared the total area of the receiver array (1.27km^2^) to the detectable area within the array (ie the summed area around each receiver with a 23.5 m radius, totaling to 0.16km^2^ for 94 receivers), as well as the total area used per individual (100% minimum convex polygon per tagged individual) and the detectable area within this area (the summed area around each receiver with a 23.5 m radius within the 100% minimum convex polygon).

### Data analyses

We performed all statistical analyses in R 4.4.0 (R Core Team 2024). First, we tested whether localization rates for each method varied across days and time of day for all tags using hierarchical generalized additive models (HGAMs) with a beta distribution using *mgcv* 1.9.1 (Wood 2011), which allows for different nonlinear relationships across different groups (Pedersen et al. 2019). Here, localization rates per method per tag was the predictor variable for separate. We fitted a global nonparametric smoothing parameter for date or hour of day, as well as factor smoothers for the parameter where the effect can vary by individual identity. All HGAMs that we fitted were checked the model for the appropriate number of basis functions (*k*), and whether the residuals were normally randomly distributed, and no assumptions were violated.

For all further analyses, we used pairs where both partners were radio-tagged and had high enough detection rates (ie comparable to expected detection rates) and home-ranges (see below) that did not include receivers on the edge of the receiver array, indicating that they spent most of their time inside the receiver array. We used continuous time movement models (CTMMs) using both the multilateration and strongest detection method to analyze space use and movement of each radio-tagged chirruping wedgebill. Localizations for each tag were fit using *ctmm* 1.2.0 (Calabrese et al. 2016) using maximum-likelihood approaches. These CTMMs account for serial autocorrelation inherent to movement data and estimate confidence intervals to space use and movement. For each tag, we visually inspected the autocorrelation structure with variograms. The best fitting model for each tag was selected based on AICc.

#### Home range size across time

Space-use for each tagged individual was calculated with the best-fitting CTMM, from which we extracted the autocorrelated kernel density estimation (AKDE) describing the utilization distribution of each individual (Fleming and Calabrese 2017). Time-dependent changes in space-use were determined by calculating the weekly AKDE of each individual, starting from September 7th, ie one day after the first individuals were radio-tagged. Weekly AKDEs were separated into 4 sections across time of day for each individual: (1) 0 to 3, (2) 3 to 6, (3) 6 to 9, and (4) 9 to 14 hours after sunrise, respectively. From each AKDE, we extracted the 95% CI home range area in km^2^ and log-transformed this to ensure model assumptions were met. Then, we fitted HGAMs with a Gaussian distribution with global nonparametric smoothing parameters for week and section of day and factor smoothers for each parameter with individual identity. Using the HGAM output, we inspected whether home range size changed after the breeding season, and all further analyses were separated between breeding season and post-breeding season.

#### Space-use overlap

To test space-use overlap, AKDEs (home ranges) of each tagged individual were calculated using the best-fitting global CTMMs including all detections between sunrise and sunset per individual, separated by breeding and post-breeding season. To assess space-use overlap, we calculated the overlap between these AKDEs for each possible dyad using the Bhattacharyya coefficient (BC), which describes the similarity between two probability distributions ranging from 0 (completely dissimilar) to 1 (identical). All possible dyads between the individuals that we tagged (*N* = 66) were classified in a dyad type: (i) pair-bonded (ii) not pair-bonded. We fitted mixed-effects beta-regression models using *glmmTMB* 1.1.9 (Brooks et al. 2017) with, as a response variable, space-use overlap, as predictor variables dyad type, breeding season (yes/no), and an interaction between dyad type and breeding season to test if pairs specifically changed their space use sharing after breeding. We removed this interaction if it was not significant based on a likelihood-ratio test (LRT). As random effects we included identity of both dyad members. This model was compared to a model excluding dyad type using a LRT. Significance of pairwise comparisons within categorical variables was determined with estimated marginal means using *emmeans* 1.10.2 (Lenth 2016). We checked for any violations of the model assumptions (residual normality and homoscedasticity) using *DHARMa* 0.4.6 (Hartig 2022) and found none.

#### Pairwise distances

To test whether partners remain in close proximity to one another and move together throughout their territory, we quantified the separation distances between pair-bonded individuals for each 15-second point using the *distance* function of *ctmm* using the best-fitting global CTMMs per breeding/post-breeding season. As a control, we compared pairwise separation distances between the focal individual at day x and their partner at day x + 1 at the same time of day, to assess whether pairs are closer to each other than expected if they were to move independently of their partner. We refer to this as the null expected distance distribution. We used this rather than simulations, since we expect chirruping wedgebills to have certain movement patterns across the day that are not captured by simulations that assume random movement within their home range. We fitted gaussian HGAMs on separation distances (mean-centred and divided by 1 standard deviation to facilitate model convergence), with global nonparametric smoothing parameters for time of day (post-sunrise) and date, including factor smoothers for each parameter where the effect can vary by pair identity, and a factor smoother for time of day including whether it was the breeding or post-breeding season. Finally, we included the categorical parameters day (x or x + 1) to test whether true separation distances differ (day x) from the null expected distance distribution (day x + 1) and breeding season (Yes/No).

#### Following behavior

We quantified how much partners follow each other continuously by identifying following events. Following events were assessed using the strongest detection method as this allowed us to categorize shared locations as receiver identities. To do so we first identified to what extent either a male or female initiated movement away from a location where the pair were previously simultaneously present. Here, we first identified movement events of the partner to a location, ie when it was most strongly detected by a different receiver than the during the previous detection with high confidence (RSS > −80). To identify following events, we first quantified all movement events of the focal individual from location A to location B and following by the partner from location A to B. If the partner followed between the initiation of movement of the focal individual from location A to the last detection of the focal individual at location B, we identified this as a following event. Then, we set a threshold at the 90th percentile of time lag between the arrival time of the focal individual and the partner at location B, as some following events had extreme time-lags due to missing detections (up to 11 hours) that could not realistically be true following events. For further analysis, we captured all following events where the partner arrived within the threshold from the arrival of the partner. Following rates were determined by taking the fraction of movement events of the partner that included a following event of the focal individual, excluding all movement events where at least one bird was not detected within the time threshold (90th percentile). We fitted a binomial HGAM on whether the focal individual followed the partner after movement events (Yes/No), with a global nonparametric smoothing parameter for time of day (post-sunrise), including a factor smoother where the effect can vary by pair identity. Finally, we included the categorical parameters sex of follower, breeding season (Yes/No), and a two-way interaction between sex and breeding season to test whether the propensity to follow a partner differs per sex and whether it was the breeding season.

### Ethics statement

Fieldwork was conducted with permission of the Macquarie University Animal Ethics Committee (reference no. 2023/012) following the Australian Code of Practice for the Care and Use of Animals for Scientific Purposes NSW Animal Research Act 1985. Banding and handling permission was issued by the Australian Bird and Bat Banding Scheme (authority no. 3788). All data collection is in accordance with the ABS/ABAB guidelines for ethical treatment of animals.

## Results

### Radio tag localization

**Fig. 1.**
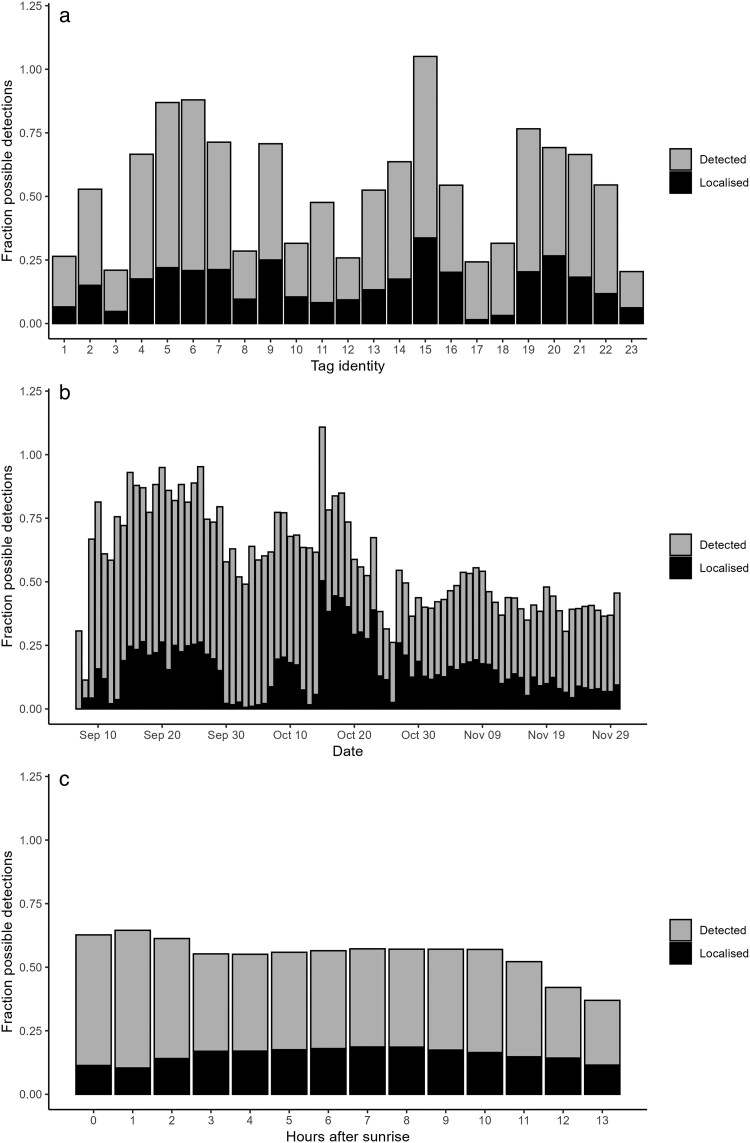
Fraction of timestamps where a bird was localized using the multilateration method (black) and strongest detection method (gray) out of all possible detections, for (a) each tagged individual, (b) each calendar date, and (c) hour of the day (in hours past sunrise). Possible detections are all timestamps between the first and last detection of the bird during daylight hours. All bars are stacked.

Localization ratios (number of localizations relative to the total number possible) of the 23 radio-tagged chirruping wedgebills varied considerably, using both the multilateration (range = 1.5 to 33.7%) and strongest detection method (range = 14.2 to 71.4%; Fig. 1a, Table S1). Localizations also varied strongly by day (strongest detection: HGAM, χ^2^ = 81.2, *P* < 0.001; multilateration: χ^2^ = 279.6, *P* < 0.001; Fig. 1b), and time of day (strongest detection: HGAM, χ^2^ = 231.4, *P* < 0.001; multilateration: χ^2^ = 74.9, *P* < 0.001; Fig. 1c). Detection rates of tags during calibration walks (Wilcoxon test: *W* = 6, *P* < 0.001), number of receivers detecting the tag (*W* = 0, *P* < 0.001), mean RSS (*W* = 1,299, *P* < 0.001), and maximum RSS (*W* = 415, *P* < 0.001) were consistently higher for elevated tags than tags at ground level (Figure S2), indicating that the detection rate and accuracy (ie average RSS) are positively affected by the height from the ground. Especially detection rate of the calibration tags was much lower, with an 8.3-fold decrease in detection rate when tags were 10 cm versus 200 cm from the ground, whereas average RSS values were not as different (max RSS: ground = −90, elevated = −73, mean RSS: ground = −103, elevated = −101).

Localization rates for the strongest detection method were much higher (mean 40.7% of total possible detections per bird, ± 8.1% s.d.) than the multilateration method, which captured only a small proportion of the possible detections (mean = 14.9%, ± 16.6% s.d.) even though there was evidence that the bird was detected. This was largely driven by the fact that often the tag was not detected by at least three receivers with a high enough RSS (≥ −80) to estimate its location. Since some radio-tagged chirruping wedgebills had consistently very low localization rates even with the strongest detection method, we selected six pairs of chirruping wedgebills where both partners were radio-tagged and had localization rates of at least 30% during the daytime. The detection rates of these birds were higher than 12.59%, which is the percentage of the total array area (1.27km^2^) in which individuals should be detectable using this method (23.5 m radius equaling to 1,735m^2^ around all 94 receivers, totaling to 0.16km^2^ which is 12.59% of the total area of the array). We also compared detection rates to the area the birds actually utilized, (area with a radius of 23.5 m around all receivers within 100% minimum convex polygon of all localizations per individual; Table S1) which often had a higher density of receivers compared to other areas within the array as receivers were preferentially placed in areas of interest to birds with more vegetation and accessible water sources. Again, the detection rates were higher than predicted localization rates within the area that the bird was actually detected (Table S1), which we elaborate on in the discussion. The 12 pair-bonded individuals selected for further analyses were tracked for between 34 and 69 days (mean = 60, ± 13 s.d), with pairs concurrently being tracked between 34 and 68 days (mean = 56, ± 16 s.d.).

Localization rates for the individuals on average did not vary across the day (Fig. 1c). The majority of the gaps in the localization of individuals were also quite short, with 58% of all of the gaps in localizations during the day being less than 10 minutes in duration, and 34% of them being less than 30 seconds (Table S2).

### Pair movement

**Fig. 2.**
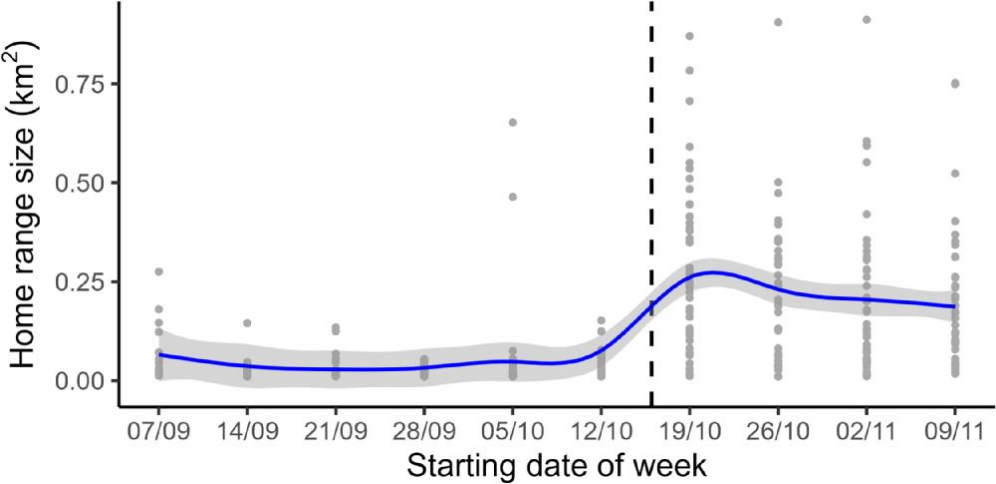
Weekly home range sizes of chirruping wedgebills (*N* = 12) between September and October 2023 calculated using the strongest detection method. Gray dots depict individual home ranges summarized by week and section of day (*N* = 428), and the line (±SE) depicts the global model predicted changes in home range size across time (in weeks). The dashed line indicates the end date of the breeding season (16/10).

We found 17 nests that were active between 11th Aug and 16th Oct 2023, with clutches of either one or two eggs (Table S3). During the period when reproductive success was monitored, five out of 17 nests produced at least one fledgling. Pair-bonded tagged birds (*N* = 12) used in the analyses were detected an average of 1,835 times per day (± 510 s.d.). Home range size varied nonlinearly by week, but became consistently larger after the last nest became inactive on Oct 16th (Fig. 2). Given that there was a population-wide ending to breeding on this date, and the strong behavioral change related to this in space use, we divided the data into breeding and post-breeding periods (before and after Oct 16th) for all further analyses. Home ranges also decreased almost linearly across time of day, although this effect was relatively small (Figure S3).

**Fig. 3.**
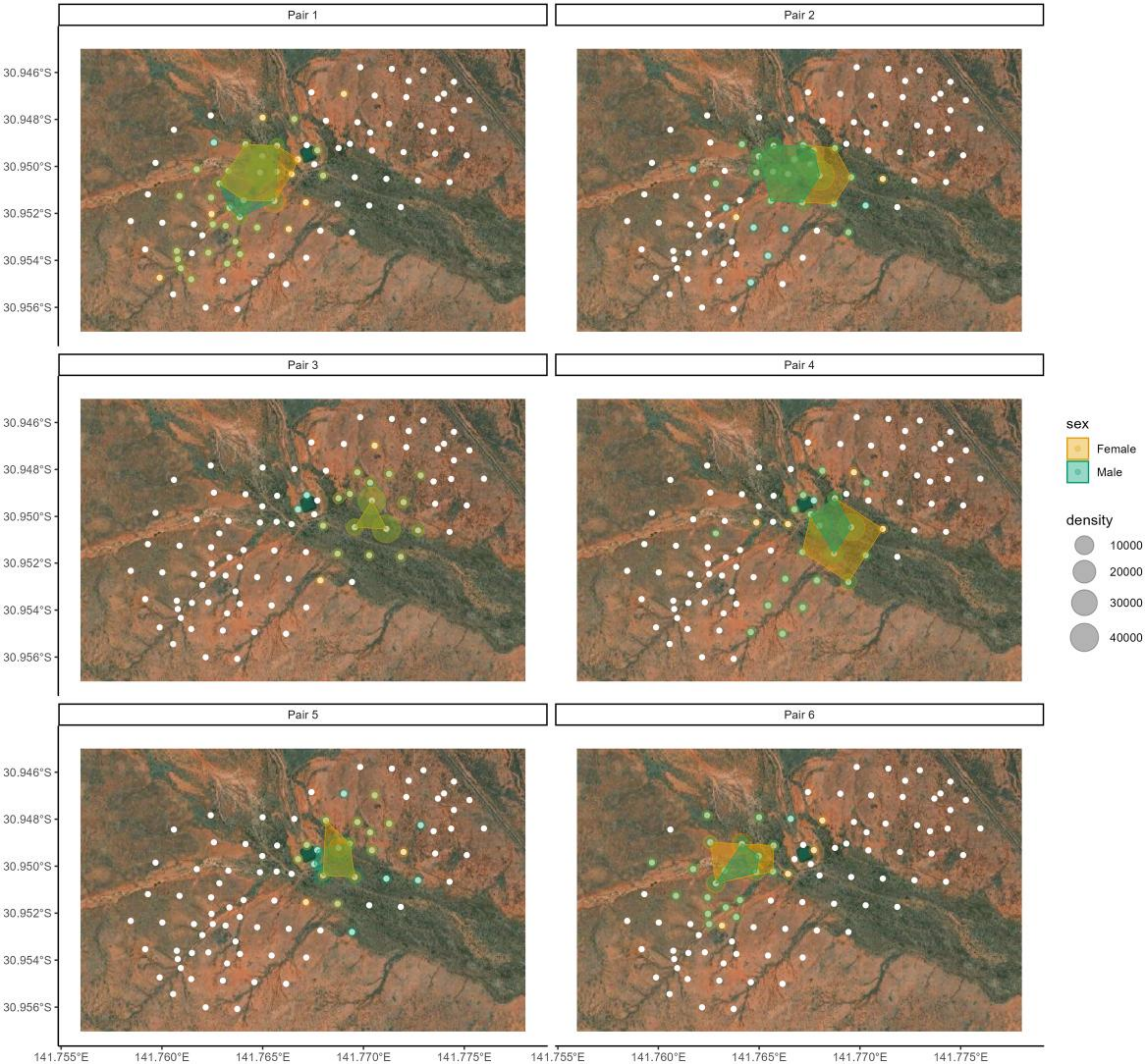
Space-use (expressed as the 95% minimum convex polygon) of pair-bonded chirruping wedgebills during the breeding season (shaded area) with detection density per radio receiver (size of circles) of each individual. Each panel indicates a unique pair-bonded dyad. Colors depict the sex of the individual (orange = female, green = male). White dots depict the radio receivers. Satellite imagery was obtained using Esri World Imagery.

**Fig. 4.**
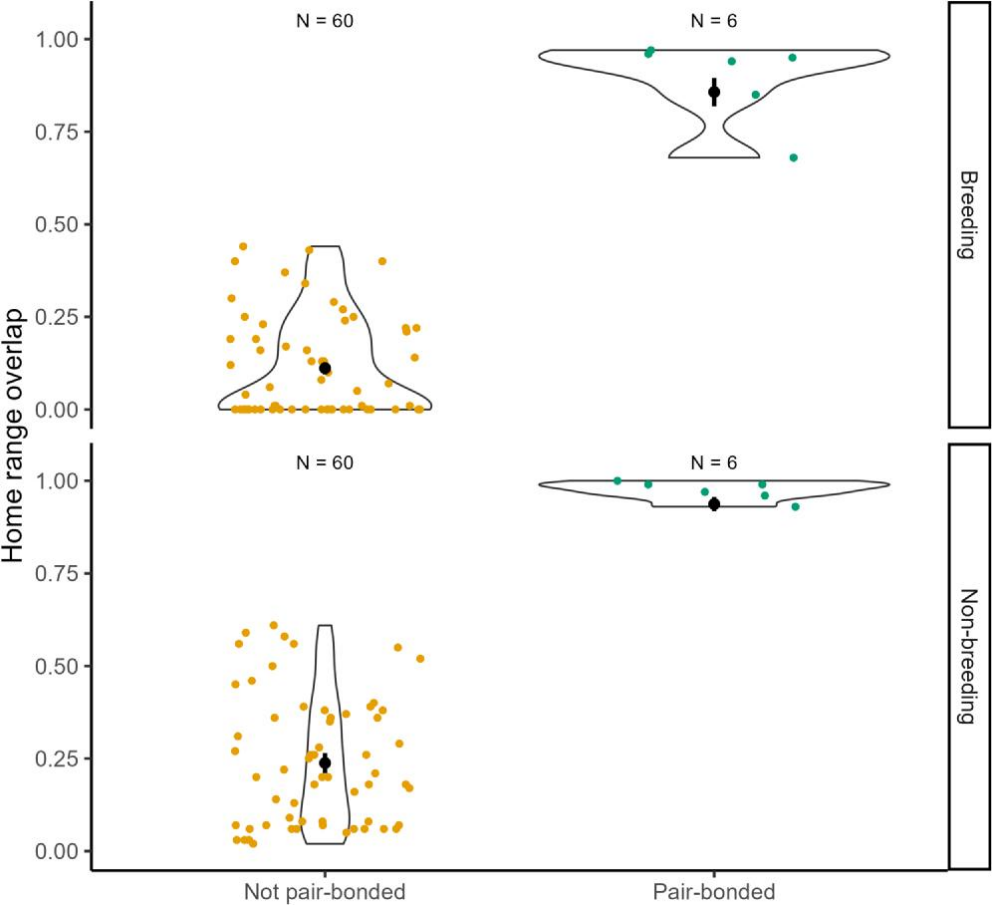
Violin plots of space use overlap (BC; Bhattacharyya coefficient) in dyads of chirruping wedgebills (*N* = 66) during the breeding and post-breeding season calculated using the strongest detection method. Dyads are classified as non-pair bonded dyads and pair-bonded dyads. Black dots and lines depict the model prediction ± SE.

Home-ranges of pair-bonded individuals covered on average 0.015 km^2^ (SE = 0.002, range 0.011 to 0.028 km^2^) during and 0.139 km^2^ (SE = 0.031, range 0.024 to 0.372 km^2^) after the breeding season (Fig. 3). The home-range overlap (BC) of pair-bonded dyads (mean ± SE = 0.93 ± 0.03, *N* = 6) was significantly larger than that of dyads that were not pair-bonded (mean ± SE = 0.18 ± 0.02, *N* = 60; Fig. 4, Table 1, LRT *P* < 0.001) for both the multilateration and strongest detection method. Although home ranges of non-pair bonded dyads overlapped (see also Figure S4), pairs still occupied distinct areas that were not or barely utilized by other pairs (Figure S5). Additionally, home-range overlap increased after the breeding season (Table 1).

**Table 1.** Beta regression of the effect of dyad-type, sex category, and breeding season on space use overlap (BC; Bhattacharyya coefficient) in dyads of chirruping wedgebills (N = 132) for both localization methods.

Method		Strongest detection				Multilateration			
Fixed effects		β	SE	z	p	β	SE	z	p
Intercept		−2.077	0.168	−12.373	<0.001	−1.512	0.187	−8.107	<0.001
Dyad type	pair-bonded	3.868	0.315	12.283	<0.001	3.378	0.354	9.557	<0.001
Breeding	no	0.912	0.156	5.862	<0.001	1.020	0.164	6.238	<0.001
**Random effects**		**SD**	**N**	…		**SD**	**N**	…	…
Focal individual ID		0.179	12	…		0.287	12	…	…
Dyad partner ID		0.266	12	…		0.300	12	…	…

Included are the model estimates (β), standard deviation (SD), and significance (z, p) of fixed effects. Random effect variances and number of levels are reported. Reference categories are dyad-type = not pair-bonded and breeding = yes.

**Fig. 5.**
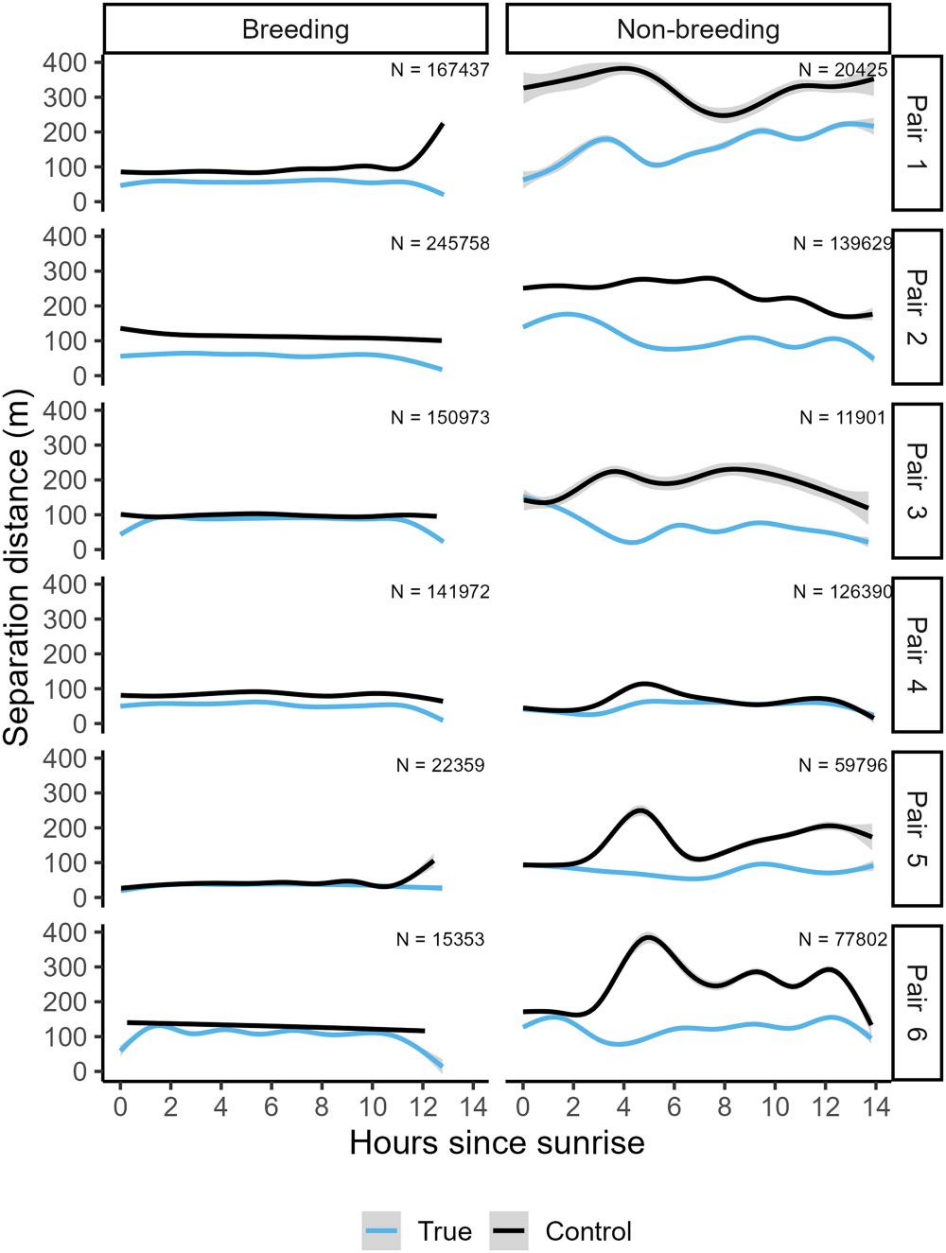
Pairwise separation distances of pair-bonded chirruping wedgebills across time after sunrise quantified using the strongest detection method. Lines depict the separation distances including the SE (shaded): true separation distances (focal day x, partner day x) and (b) the control separation distances (focal day x, partner day x + 1).

Pairwise separation distances across all pairs averaged (± s.d.) on 70.5 m ± 65.6 m but varied substantively across pairs (mean per pair: 54.4 to 90.5 m, SE = 3.84) using the multilateration method. Since multilateration does not allow for highly precise localizations estimates, the deviation in accuracy of localizations (median error 35 m) of both partners combined may inflate true pairwise separation distances. The strongest detections of partners was often at the same receiver during (55.7% ± 18.3% of all simultaneous detections across all pairs) and after breeding (70.3% ± 11.8%). The HGAMs indicated that separation distances of the null expected distance distribution (day x + 1) were significantly higher than true separation distances (day x; strongest detection: β = 0.34, SE = 0.002, *P* < 0.001; multilateration: β = 0.28, SE = 0.003, *P* < 0.001; Fig. 5), indicating that pairs moved closer to each other than expected if movement was independent from their partner. Separation distances were significantly higher during than after breeding using the strongest detection method (β = 0.37, SE = 0.023, *P* < 0.001) but not the multilateration method (β = −0.05, SE = 0.029, *P* = 0.086). We also found that separation distances were affected by time of day (strongest detection: *F* = 59.9, *P* < 0.001, multilateration: *F* = 17.6, *P* < 0.001) and date (strongest detection: *F* = 515.1, *P* < 0.001, multilateration: *F* = 263.1, *P* < 0.001) when controlling for pair identity.

**Fig. 6.**
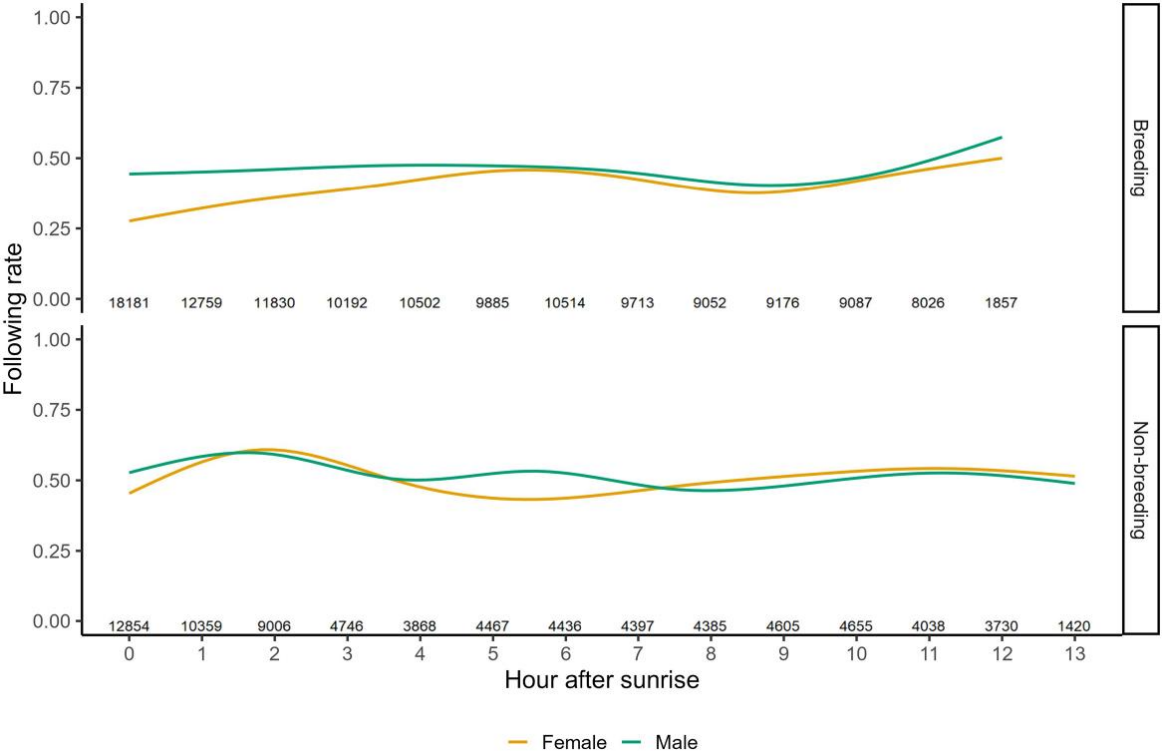
Rate of following of the focal bird after their partner initiated movement of pair-bonded chirruping wedgebills (*N* = 12) per separated by sex across time of day (hours after sunrise) during and after the breeding season using the strongest detection method. Numbers at the bottom of the plot depict sample sizes of partner-initiated movement during the time of day.

Although males were detected more often than the female (Table S4), both partners were detected in on average 40.5% of all detections within a pair-bond, of which an average of 23.6% detections was by the same receiver. Movement initiations away from a shared receiver location were equal between males and females (both on average 42.9%; Table S5), and in some cases both partners moved away from a shared location simultaneously (on average 14.2%). Of all following events, 90% happened within a span of 10 minutes, which was used as a threshold to remove extreme outliers (Figure S6). Thus, any movement initiation made by the follower after 10 minutes were not seen as following events, as we cannot assume this movement was initiated as a response to their partners' movement. Following rates were dependent on time of day when controlling for pair identity (HGAM, χ^2^ = 4,891, *P* < 0.001), and an interaction between sex of the follower and whether it was the breeding season (Fig. 6). Specifically, males (47.9% of 68.3k movements, ± 19.5% s.d., range = 26.2 to 83.4%) followed their partner more often than females followed the male (36.6% of 62.5k movements, ± 15.2% s.d., range = 17.2 to 59.7%) during breeding. Both sexes increased their following rates after breeding, and whilst males (50.5% of 42.3k movements, ± 8.2% s.d., range = 37.7 to 58.6%) still followed their partner more often than females followed the male (44.2% of 34.6k movements, ± 13.7% s.d., range = 20.9 to 59.5), this difference was smaller than during breeding (β = −0.20, SE = 0.02, *P* < 0.001).

## Discussion

Here we show that chirruping wedgebills have high spatial pair cohesion both during and after the breeding season. Using automated radiotracking, we show that home ranges increased in size after the breeding season. This, however, did not affect home range overlap: the space use of partners was nearly identical both during and after the breeding season, and had a much greater overlap than non-pair bonded dyads. Not only did partners share the same area, but they also remained in consistent proximity to one another across time of day during and after the breeding season. Partners also followed each other consistently, with high occurrence of following behavior of a partner when one individual moved to a new location. Following was more often displayed by males and occurred more often after the breeding season. Overall, socially monogamous pairs of territorial chirruping wedgebills display very high and consistent levels of spatial cohesion both during and outside of the reproductive period.

We found that localizations of individuals using the multilateration method were relatively precise but temporally limited, making this method less appropriate for the assessment of continuous patterns of movement for birds that spend substantial time on the ground, leading to fewer detections by multiple receivers. We frequently observed chirruping wedgebills foraging on the ground for insects, and they often walked rather than flew when moving short distances between foraging locations. Detection rates and signal strength were lower when tags were near the ground (in our trials with tags off birds), suggesting that the foraging habits impaired the detections of this species, and we thus may have obtained lower coverage of their movement when foraging. In denser vegetation, detection rates and accuracy using multilateration have been shown to be low, presumably due high attenuation by the vegetation (Tyson et al. 2024b). Moreover, we show that the height of the tag during calibration walks affected the likelihood of detection as well as detection strength, meaning that distance estimates are strongly influenced by the height above ground of the bird. This is consistent with other automated radiotracking studies (Ward and Raim 2011; Rueda-Uribe et al. 2024) and other tracking techniques such as ATLAS (Toledo et al. 2020; Beardsworth et al. 2022). To determine behaviors requiring high detection precision (eg ground foraging), receivers would need to be placed in an even higher density array to ensure detection rate and precision are optimized. Additionally, other technologies, such as accelerometers (Brown et al. 2013; Christensen et al. 2025), can be useful in inferring foraging behavior and other behaviors affecting detections.

The strongest detection method, where we identified the locations of birds using the strongest detection of a receiver, yielded much higher localization rates over time, than the multilateration method discussed above. Surprisingly, the detection rates tended to be higher than the expected detectable area would predict if chirruping wedgebills were to use their home ranges homogenously. This can be explained by (1) the birds spending relatively more time in proximity to the receivers than other areas or (2) the RSS being consistently stronger than expected at larger distances from the receivers. (1) As receivers are not placed fully homogenously in the array, but occur in higher density along creek lines, water sources, and areas of dense vegetation, birds may be more often in proximity to receivers as they may prefer these areas. (2) The RSS-distance relationship can be affected by many variables, such as the height of the tag (as discussed above) and orientation of the tag and receiver (Whitehouse et al. 2007). For instance, when at greater height, such as perched on top of trees which chirruping wedgebills were frequently observed doing during this study, the RSS may be stronger compared to the expected RSS from calibrations at 1.5 m. Overall, due to the intensive nature of our data collection design (with tags being detected every 15 seconds for months) our large dataset still yielded high enough detection rates and total numbers of detections per individual to make meaningful inferences about their movement and pair cohesion using a combination of the two analytical approaches (strongest detection and multilateration).

We defined breeding and post-breeding as a population-wide metric, since behavior may be strongly influenced by breeding of conspecifics in the same area. Especially a fundamental behavior like movement may be sensitive to this, even more so given the territoriality of this species. Territoriality is often strongly influenced by the stage of the breeding cycle (Odum and Kuenzler 1955; Finck 1990; Class and Moore 2011; Reid et al. 2022), and in turn may be strongly related to movement (Finck 1990; Naguib et al. 2022). Indeed, territoriality, and thereby movement, should be strongly related to the social environment and structure (Snijders and Naguib 2017). Our results are in line with this notion, as we found a strong change in space use after the last nest became inactive.

The home range area for the male and female within a pair were nearly identical, supporting the notion of these birds being territorial, although some home range overlap with other individuals outside the pair-bond exists. This home range overlap outside the pair is unsurprising, as it is likely driven by extraterritorial forays, which define the home range along with the territory boundaries (Bircher et al. 2020) and largely determine social associations across territory boundaries (Snijders et al. 2014). Besides sharing highly similar areas, chirruping wedgebill partners tend to be in close proximity to each other consistently, both during and after the breeding season, indeed suggesting that pairs stay together beyond one breeding event. Although shared territoriality alone can explain the strong home range overlap, the continuously high levels of spatial proximity indicate that partners are likely coordinating their movement to each other. Interestingly, consistent pairwise proximity is often used to identify social relationships eg by recording social networks (see Krause et al. 2011; Farine 2015), rather than explicitly studied. We found that chirruping wedgebill partners follow each other at high rates, showing that the spatial proximity is driven by influence of one individual on another (Strandburg-Peshkin et al. 2018). This results in active decision-making (following your partner) rather than passive processes, such as a lack of movement by both partners that happen to be in the same area with a certain resource. Strikingly, partners tend to follow each other more after the breeding period, when there is no need for nest attendance and when home ranges are considerably larger. During breeding, nest attendance is a crucial part of nesting success, meaning partners may need to consistently alternate nest attendance (eg Bebbington and Hatchwell 2016). This, in turn, might result in lower rates of following behavior. We highlight that measures of proximity and following behavior provide useful insights on the spatial coordination within a pair beyond home-range overlap.

Consistent proximity and coordination in movement may benefit socially monogamous pairs in multiple ways. Especially in species with biparental care, strong pair cohesion can improve cooperation to raise offspring. Continuous close contact enables partners to coordinate nest visiting and attendance during incubation and provisioning, resulting in higher reproductive success (Mariette and Griffith 2012; Bebbington and Hatchwell 2016; Tyson et al. 2017; Wojczulanis-Jakubas et al. 2023). It also prevents over-investment in parental care of one partner during the reproductive event, which would be detrimental for potential future reproductive events for both partners if they would remain pair bonded (Mariette and Griffith 2015). This means that coordination will not only benefit the current reproductive attempt, but also potential future reproductive success of the pair. Maintaining consistent close contact with a partner also may increase readiness to initiate breeding. For example, captive zebra finches that form more stable partnerships initiate breeding faster (Adkins-Regan and Tomaszycki 2007; Maldonado-Chaparro et al. 2021). In great tits, individuals that meet earlier after the breeding season initiate breeding faster, and produce larger clutches (Culina et al. 2020). Especially in unpredictable and harsh environments like the habitat of the chirruping wedgebill, readiness to breed may be a crucial factor determining breeding success.

Benefits of strong pair cohesion in socially monogamous pairs extend outside the reproductive context. Partners that have strong spatial cohesion may increase their efficiency in locating and exploiting ephemeral resources. Here, individuals in strong partnerships attain the food sources faster as they are paying attention to (Dall and Griffith 2014) and helping (Mariette and Griffith 2015) one another. Indeed, socially monogamous partners can plastically adjust their foraging coordination to reproductive demands—such as clutch size—and pairs with high foraging coordination yield reproductive benefits (Mariette and Griffith 2015). Additionally, there may be a reduction in predation likelihood, as there is more predator awareness and potentially a lowered attraction of predators to non-solitary prey that are together with their partner (Beauchamp 2002). In the context of territorial species, a strong cohesion with a co-owner of the territory, in this case the partner, may result in more effective territory defense. Chirruping wedgebills produce antiphonal duets, whereby the male and female produce a joint song with alternating syllables (Austin et al. 2019). These types of duets have often been related to pair quality and territory defense (Hall 2004, 2009; Dahlin and Benedict 2014), where the rate and coordination of the male and female song exemplifies their quality (Hall 2000). Being in consistent close proximity enhances duetting rate and duet length, as partners are more likely to answer each other's call (Logue 2007). Thus, consistent proximity may allow more effective signaling against intruders through duetting. All this coincides with the persistently high home range overlap, continuous close proximity, and high following rates that we found in the chirruping wedgebills after breeding. In fact, chirruping wedgebills follow each other at higher rates during the non-reproductive period compared to the reproductive period. This is likely due to the absence of movements related to attendance and alternation at a nest, meaning partners can consistently stay together as they move.

## Conclusion

This study continuously tracking partner movements, shows extremely high spatiotemporal synchrony within socially monogamous pairs of a territorial passerine both during and after the breeding season. This adds to existing findings of strong behavioral synchrony in movement in socially monogamous passerines (Baldan and van Loon 2022; Tyson et al. 2024a), and shows that spatial cohesion persists outside the reproductive period. We suggest that cooperation within the partnership, rather than sexual conflict, largely drives these behaviors. Indeed, multiple studies have shown that there are fitness benefits associated with maintaining a partnership (eg van de Pol et al. 2006; Sánchez-Macouzet et al. 2014; Ihle et al. 2015; D’Amelio et al. 2024). We add to this by highlighting that paying attention to and moving with a partner is likely to be very important outside the breeding context, as cooperation and coordination are still highly valuable for functions other than parental care (Griffith 2019). Still, very few studies have explored association strength outside of the breeding context, and to our knowledge none using fine-scale movement in the wild. Overall, the strength of the association within the pair as a result of the decisions made by both partners is a crucial factor in these partnerships. We suggest that space-use is an important and appropriate metric to quantify association strength in the partnerships of mobile animals.

## Supplementary Material

araf130_Supplementary_Data

## Data Availability

Analyses reported in this article can be reproduced using the data and code provided by Speelman et al. (2025).
