# Supplementary material for: High spatial pair cohesion during and after breeding in a socially monogamous territorial passerine
Source: Behav Ecol. 2025 Nov 6;36(6):araf130. doi: 10.1093/beheco/araf130 (PMC12645101; doi:10.1093/beheco/araf130)
Supplement: araf130_Supplementary_Data [file araf130_supplementary_data.pdf]

SUPPLEMENTARY FILE FOR:

Speelman, Frigg; Tyson, Chris; Naguib, Marc; Griffith, Simon (2025). High spatial pair cohesion during and after breeding in a socially monogamous territorial passerine. *Behavioral Ecology*.

SUPPLEMENTARY FIGURES

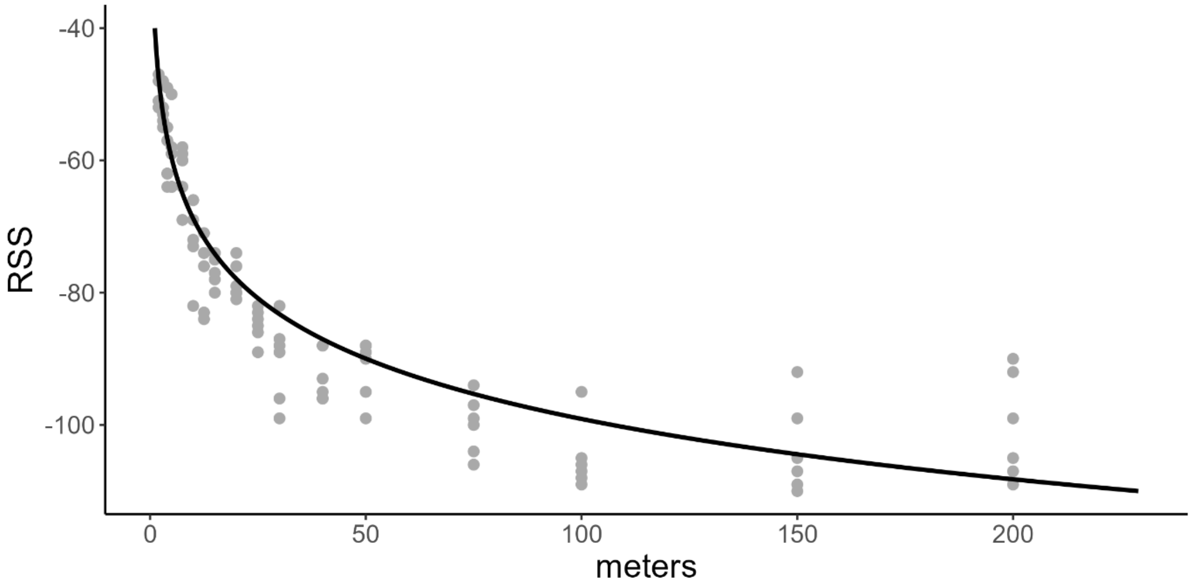

Figure S1. RSS versus distance curve (black) including RSS values of six radio tags held at set distances (grey points).

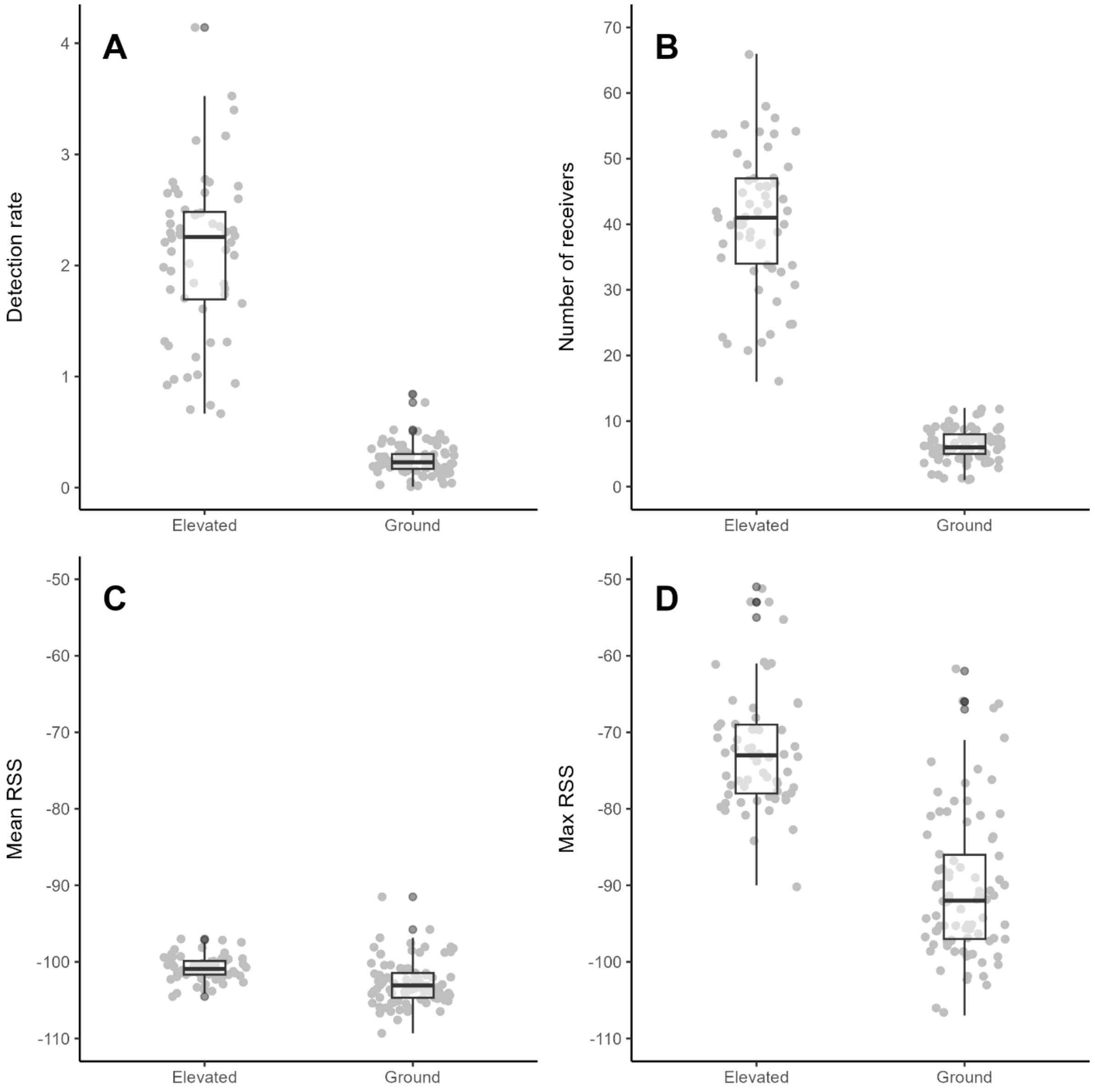

Figure S2. Detections of 6 tags mounted on poles at 200cm (Elevated) and 10cm (Ground) within 2 minute-intervals. Indicated are (A) detection rates, (B) the number of receivers detecting the tag, (C) the mean RSS value of all detections per tag and (D) the maximum RSS value of all detections per tag.

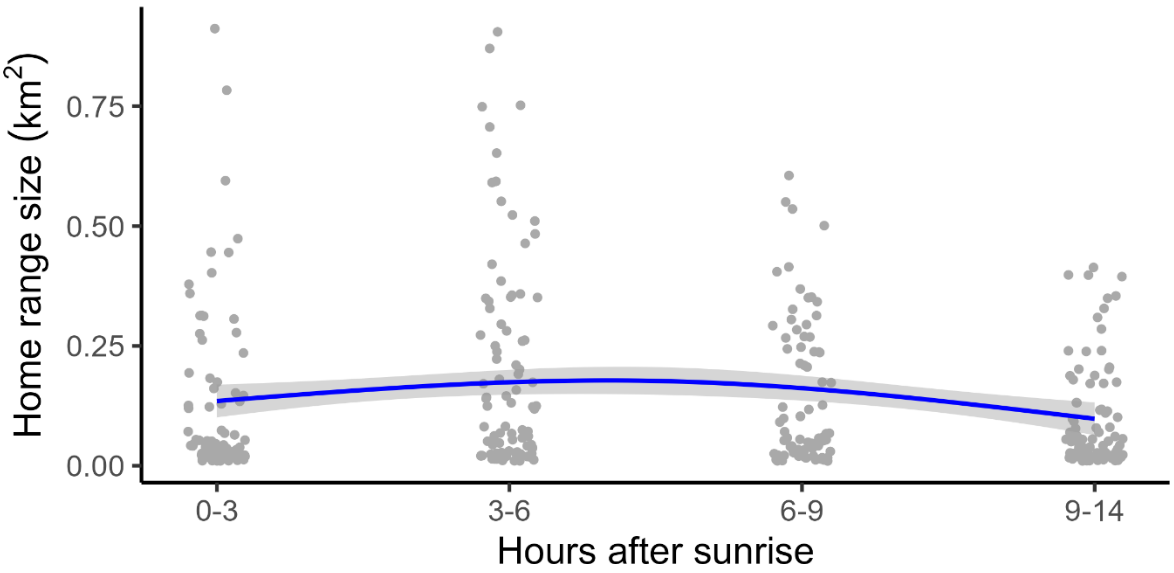

Figure S3. Home range sizes of chirruping wedgebills (*N*=12) as calculated by the strongest detection method across four intervals during the day. Grey dots depict individual home ranges summarised by week and interval (*N*=428), and the blue line depicts the global model predicted changes in home range size across time (hours after sunrise).

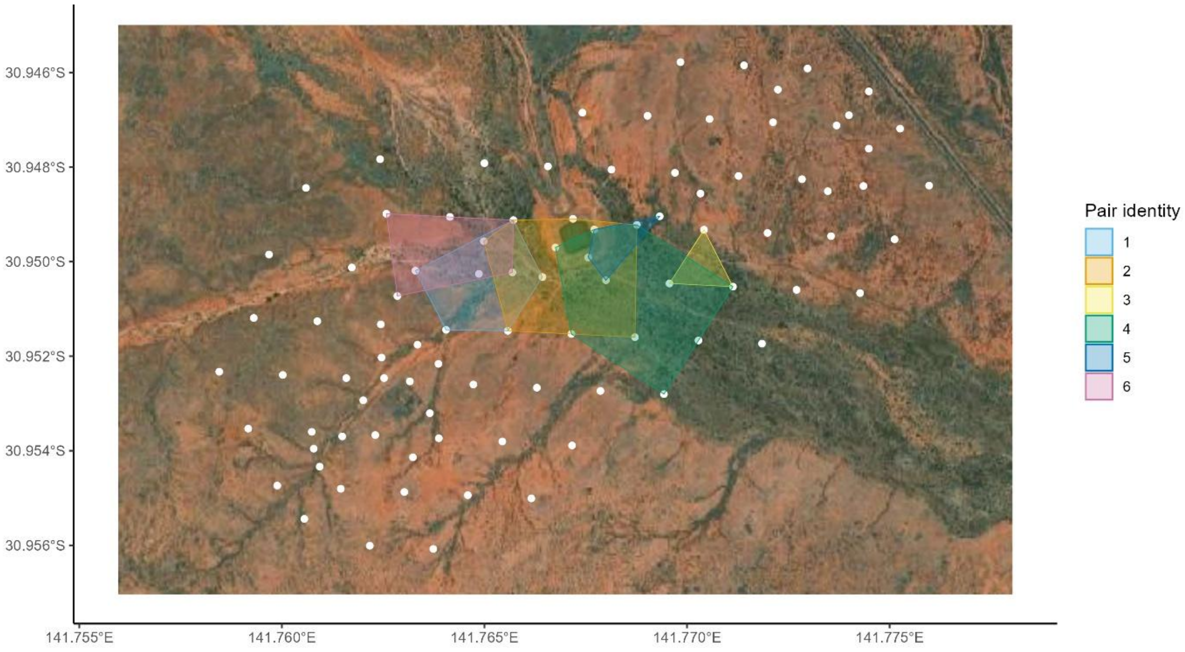

Figure S4. Pair-level space-use (expressed as the 95% minimum convex polygon) of chirruping wedgebills during the breeding season (shaded area) using the strongest detection method. Each polygon indicates the total space-use of a pair-bonded dyad. Colours depict the identity of the pair, white dots depict the radio receivers. Satellite imagery was obtained using Esri World Imagery.

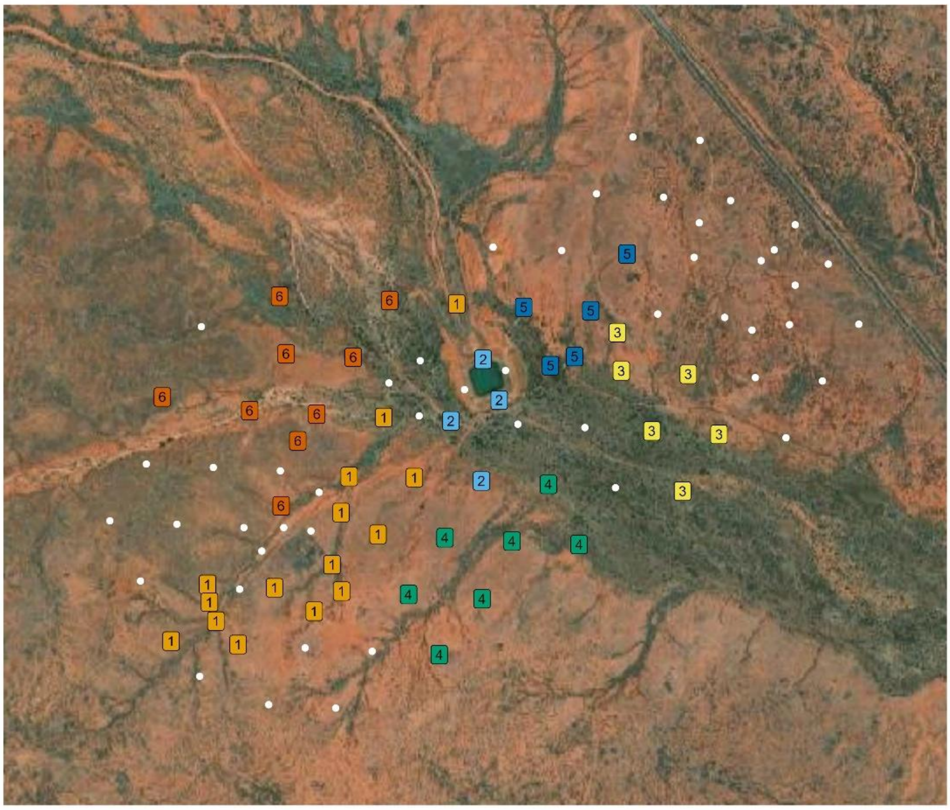

Figure S5. Areas of exclusivity per pair of chirruping wedgebills (N=6) where nodes are indicated that have >=80% of detections coming from a single pair (coloured text boxes) using the strongest detection method. White dots depict the radio receivers. Satellite imagery was obtained using Esri World Imagery.

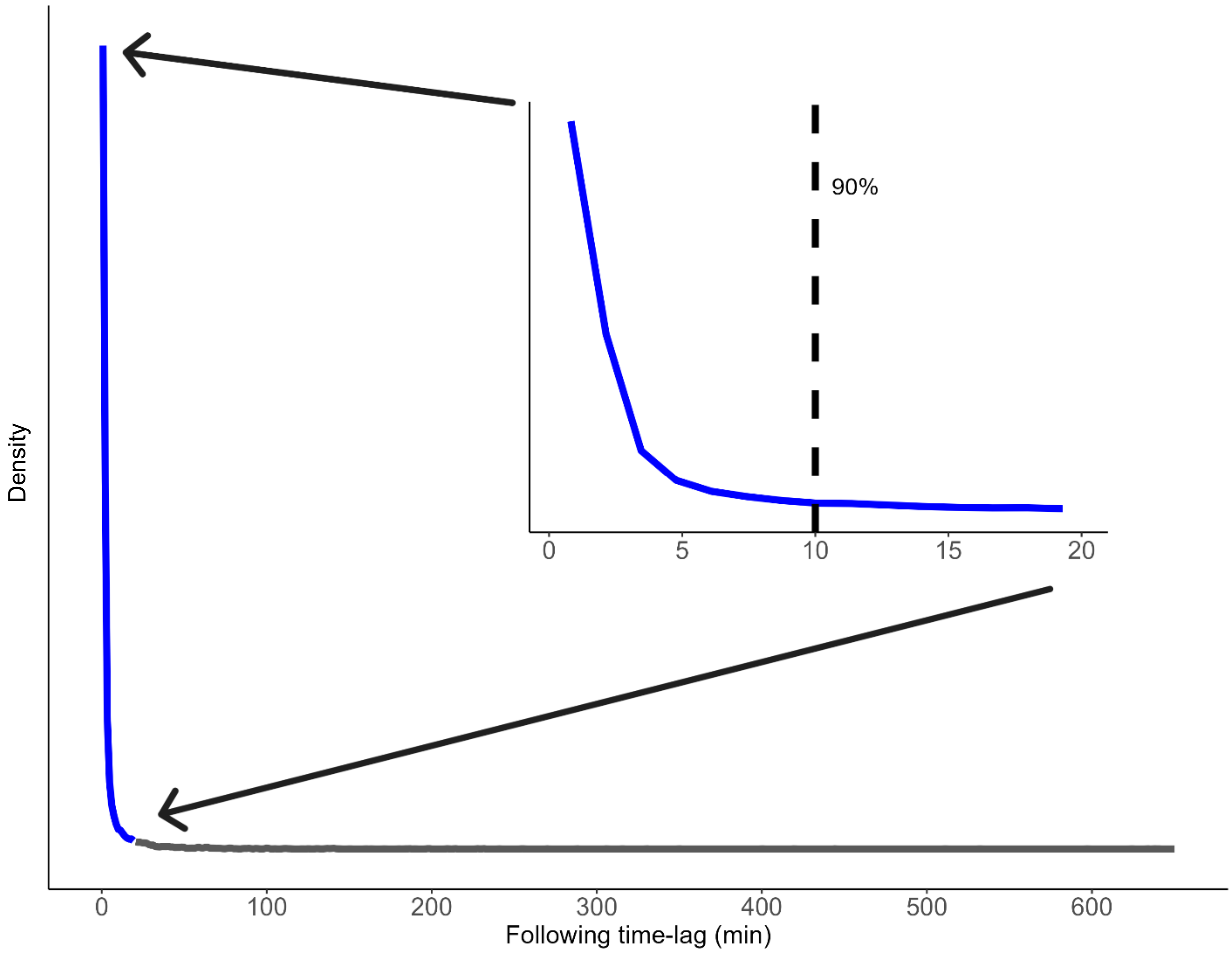

Figure S6. Density plots of time-lag between arrival time of the focal individual and the arrival of their partner (the follower) from location A to B using the strongest detection method. Included are all time-lags, between 15 seconds and 20 minutes (in blue) and 20 and 673 minutes (in grey). In the smaller plot, the density plot of the 15-second to 20-minute time lags is given, corresponding to the blue line in the main plot. The dashed line represents the 90th percentile of data, corresponding to a time lag of 10 minutes.

SUPPLEMENTARY TABLES

Table S1. Number of localisations, localisation-rates, and detectable area per radio-tagged chirruping wedgebill. Included are the possible localisations (i.e. all time intervals between first moment of detection and last moment of detection), the actualised detections using the multilateration and strongest detection method, and the percentage of actualised localisations per method. The detectable area indicates the total area (in km^2) in which birds were localised (100% MCP of localisations), the detectable area (in km^2) within this total area (23.5m radius around receivers that detected the bird), and the percentage of the total area that was detectable. In bold are the tags of pair-bonded chirruping wedgebills that were used for further analysis (both individuals had localisation-rates of at least 30% using the strongest detection method).

	Possible	Multilateration		Strongest detection		Detectable area		
Tag identity	*N*	*N*	%	*N*	%	Total km^2	Possible km^2	%
1	237719	15533	6.5	47285	19.9	*NA*	*NA*	*NA*
**2**	**272171**	**40983**	**15.1**	**102828**	**37.8**	**0.13**	**0.02**	**17.5**
3	259739	12301	4.7	42190	16.2	*NA*	*NA*	*NA*
**4**	**263749**	**46227**	**17.5**	**129398**	**49.1**	**0.17**	**0.02**	**12.4**
5	273877	60151	22.0	177913	65.0	*NA*	*NA*	*NA*
**6**	**301783**	**62815**	**20.8**	**202652**	**67.2**	**0.19**	**0.03**	**15.8**
**7**	**225925**	**47912**	**21.2**	**113232**	**50.1**	**0.13**	**0.02**	**16.4**
8	208938	19970	9.6	39579	18.9	*NA*	*NA*	*NA*
**9**	**293883**	**73414**	**25.0**	**134369**	**45.7**	**0.24**	**0.04**	**17.9**
10	263207	27495	10.4	55554	21.1	*NA*	*NA*	*NA*
**11**	**269490**	**22192**	**8.2**	**106183**	**39.4**	**0.15**	**0.02**	**14.0**
12	262871	24435	9.3	43450	16.5	*NA*	*NA*	*NA*
13	267505	35544	13.3	104824	39.2	*NA*	*NA*	*NA*
**14**	**253840**	**44364**	**17.5**	**117147**	**46.1**	**0.15**	**0.03**	**17.0**
**15**	**329241**	**110804**	**33.7**	**235035**	**71.4**	**0.27**	**0.03**	**12.7**
**16**	**170774**	**34399**	**20.1**	**58513**	**34.3**	**0.10**	**0.02**	**19.6**
17	170560	2584	1.5	38779	22.7	*NA*	*NA*	*NA*
18	203618	6478	3.2	57802	28.4	*NA*	*NA*	*NA*
**19**	**268819**	**54628**	**20.3**	**151260**	**56.3**	**0.17**	**0.03**	**15.6**
**20**	**170388**	**45311**	**26.6**	**72586**	**42.6**	**0.09**	**0.02**	**22.1**
**21**	**296466**	**54068**	**18.2**	**143044**	**48.2**	**0.30**	**0.05**	**16.3**
22	116759	13735	11.8	49904	42.7	*NA*	*NA*	*NA*
23	122681	7608	6.2	17458	14.2	*NA*	*NA*	*NA*

Table S2. Absent localisations using the multilateration method and strongest detection method for all 23 radio-tagged chirruping wedgebills. Absent localisations are categorized by length of the gap (time interval that has no localisation per individual). Included are the number of gaps per method, and the percentage of time these gaps account for the total time of all gaps.

	Multilateration		Strongest detection	
Length of gap	*N* gaps	time %	*N* gaps	time %
≤30sec	2301411	6.6	20488200	34.4
≤1min	1782540	5.1	4106985	6.9
≤2min	1475393	4.2	2593665	4.4
≤5min	2764230	7.9	3502770	5.9
≤10min	3672555	10.5	4234095	7.1
≤30min	8890290	25.4	8917215	15.0
≤1hr	5794710	16.6	5464020	9.2
≤2hr	4627050	13.2	4090485	6.9
≤4hr	2319315	6.6	2889285	4.9
>4hr	1327680	3.8	3202605	5.4
Total	34955174	100.0	59489325	100.0

Table S3. Nests of chirruping wedgebills in Gap Hills between Aug 11th and Oct 16th including the finding date and ending date, whether they were successful (at least one chick fledged). Pair identity is indicated for radiotagged pairs.

Nest nr.	Find date	End date	Success	Pair identity
1	2023/08/11	2023/08/30	0	1
2	2023/08/12	2023/08/24	0	5
3	2023/08/12	2023/08/30	1	
4	2023/08/13	2023/08/24	0	
5	2023/08/17	2023/09/01	1	
6	2023/08/18	2023/09/03	0	
7	2023/08/18	2023/09/07	1	4
8	2023/08/18	2023/09/09	0	
9	2023/08/24	2023/09/09	0	2
10	2023/08/29	2023/09/22	0	
11	2023/08/31	2023/09/29	1	6
12	2023/09/01	2023/09/03	0	
13	2023/09/01	2023/09/09	0	3
14	2023/09/12	2023/09/16	0	
15	2023/09/27	2023/10/13	0	
16	2023/09/30	2023/10/13	0	
17	2023/09/16	2023/10/16	1	

Table S4. Percentage of 15-second intervals with detections per pair of chirruping wedgebills using the strongest detection method. Percentages are indicated for where only one partner was detected, separated by sex, or both partners were detected, separated by whether they were detected by the same radio receiver (same location) or a different receiver (different location).

	One partner %		Both partners %	
Pair identity	Male only	Female only	Same location	Different location
1	29.2	29.5	22.0	19.3
2	25.2	16.9	31.9	26.0
3	26.4	31.4	19.0	23.2
4	29.8	19.6	38.8	11.8
5	58.0	19.0	16.1	6.8
6	22.5	49.9	13.6	14.0
Average	31.9	27.7	23.6	16.9

Table S5. Percentage of initiated movements away from a shared location per pair of Chirruping wedgebills. Included are all events where both partners were at the same location, and indicated is the percentage of events where the focal individual (male, female, or both partners) was the first to show up at a different location. Included are the averaged initiated movement rates.

Pair identity	Male	Female	Both
1	45.2	38.9	15.9
2	48.4	41.3	10.3
3	43.5	46.0	10.5
4	38.5	48.9	12.6
5	40.5	48.7	10.8
6	41.2	33.7	25.1
Average	42.9	42.9	14.2
